# Al-Doped Octahedral Cu_2_O Nanocrystal for Electrocatalytic CO_2_ Reduction to Produce Ethylene

**DOI:** 10.3390/ijms241612680

**Published:** 2023-08-11

**Authors:** Sanxiu Li, Xuelan Sha, Xiafei Gao, Juan Peng

**Affiliations:** State Key Laboratory of High-Efficiency Utilization of Coal and Green Chemical Engineering, College of Chemistry and Chemical Engineering, Ningxia University, Yinchuan 750021, China; sanxiu2023@163.com (S.L.); shaxuelan2023@163.com (X.S.); gaoxiafei0204@163.com (X.G.)

**Keywords:** electrocatalysis, electronic structure, faradaic efficiency, ethylene

## Abstract

Ethylene is an ideal CO_2_ product in an electrocatalytic CO_2_ reduction reaction (CO_2_RR) with high economic value. This paper synthesised Al-doped octahedral Cu_2_O (Al–Cu_2_O) nanocrystal by a simple wet chemical method. The selectivity of CO_2_RR products was improved by doping Al onto the surface of octahedral Cu_2_O. The Al–Cu_2_O was used as an efficient electrocatalyst for CO_2_RR with selective ethylene production. The Al–Cu_2_O exhibited a high % Faradic efficiency (FE_C2H4_) of 44.9% at −1.23 V (vs. RHE) in CO_2_ saturated 0.1 M KHCO_3_ electrolyte. Charge transfer from the Al atom to the Cu atom occurs after Al doping in Cu_2_O, optimizing the electronic structure and facilitating CO_2_RR to ethylene production. The DFT calculation showed that the Al–Cu_2_O catalyst could effectively reduce the adsorption energy of the *CHCOH intermediate and promote the mass transfer of charges, thus improving the FE_C2H4_. After Al doping into Cu_2_O, the center of d orbitals shift positively, which makes the d–band closer to the Fermi level. Furthermore, the density of electronic states increases due to the interaction between Cu atoms and intermediates, thus accelerating the electrochemical CO_2_ reduction process. This work proved that the metal doping strategy can effectively improve the catalytic properties of Cu_2_O, thus providing a useful way for CO_2_ cycling and green production of C_2_H_4_.

## 1. Introduction

The increased CO_2_ emissions in the atmosphere result in a serious greenhouse effect and the elevated sea level [1,2]. The electrochemical CO_2_ reduction reaction (CO_2_RR) is a promising strategy to mitigate the global warming and energy crisis while transforming CO_2_ into fuels and chemical feedstocks [3,4,5]. It can use clean electric energy generated by renewable solar and wind energy to drive the conversion of CO_2_ under mild conditions [6,7]. Electroreduction of CO_2_ in molten salts, also called molten salt carbon capture and electrochemical transformation (MSCC-ET), is an advanced method which can capture carbon dioxide from the atmosphere or flue gases [8,9,10,11]. The reduction products of CO_2_RR include CO [12,13,14], HCOOH [15,16,17,18], alcohols [19,20,21], and various hydrocarbons [22,23,24]. Among them, C_2_H_4_ has attracted more and more attention due to its high energy density. Furthermore, C_2_H_4_ is essential in producing various plastics, solvents, and cosmetics in the chemical industry [25].

Up to now, Cu-based materials can electrocatalytic convert CO_2_ into C2/C2+ products. Among reported Cu-based catalysts, Cu_2_O nanocrystal has attracted much attention due to their electrocatalytic activity and high selectivity toward C_2_H_4_. To improve the CO_2_RR performance of Cu_2_O, great efforts have been made on its structural design. Metal ions can be used as structure-guiding agents to optimize the micro-structure of nanocrystals [26]. Cu_2_O nanoparticles (Cu_2_O NPs) exhibit good performance for CO_2_RR, possibly because the low coordination Cu^+^ on the surface contributes to C-C coupling, thus promoting the production of C_2_H_4_ [27]. Other strategies, including crystal facet controlling [28], defect engineering [29,30], alloying [31], valence adjustment [32], and surface molecular modification [24], have been employed to improve the electrocatalytic performance of CO_2_RR to produce C_2_H_4_. For example, Shang et al. [33] have designed a core-shell Cu@Cu_2_O catalyst on which a thin layer of natural oxide grows on the surface under environmental conditions. The synergistic effect between Cu^+^ and Cu^0^ on the Cu@Cu_2_O surface helps to improve its efficiency and selectivity for C2 products. Ning et al. [34] reported the preparation of Cu_2_O/nitrogen-doped carbon shell (Cu_2_O/NCS) composite and its application in CO_2_ electroreduction to selective formation of C_2_H_4_. However, copper-based catalysts still face many problems, such as inevitable competitive hydrogen evolution reaction (HER), complex reaction mechanisms diversification of products, and low selectivity of target products [35]. Therefore, it is of huge challenge to design CO_2_RR electrocatalysts with high activity, selectivity, and satisfied stability.

This work proposed an effective strategy to improve the CO_2_RR activity by doping Al on the surface of octahedron Cu_2_O nanocrystals. Al-doped Cu_2_O (Al–Cu_2_O) was used as an effective electrocatalyst for selective ethylene production by CO_2_RR. Al–Cu_2_O exhibits a high Faraday efficiency (FE_C2H4_) of 44.9% at −1.23 V (vs. RHE). During the coupling process of *CHCOH intermediate, the Al–Cu_2_O catalyst can significantly reduce the free energy and promote the formation of C_2_H_4_. It can also inhibit the occurrence of HER side reaction. Therefore, the doping strategy is beneficial for the adsorption of intermediates to reconstruct the internal stable state of Cu_2_O, thus improving the activity and selectivity of CO_2_ conversion to ethylene.

## 2. Results and Discussion

### 2.1. Morphology and Structure Analysis

**Figure 1:**
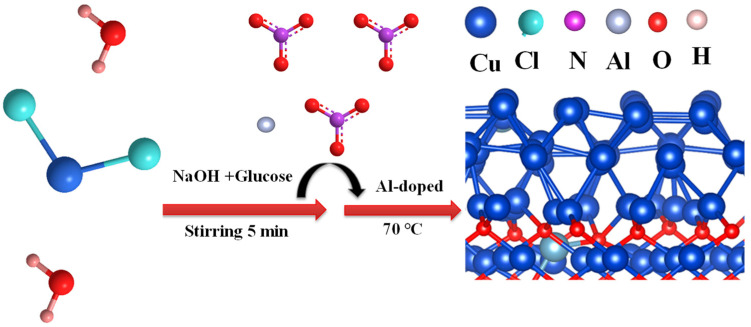
Schematic illustration of the fabrication process for Al–Cu_2_O–X (X = 2, 3, 4, 5, 6).

**Figure 2:**
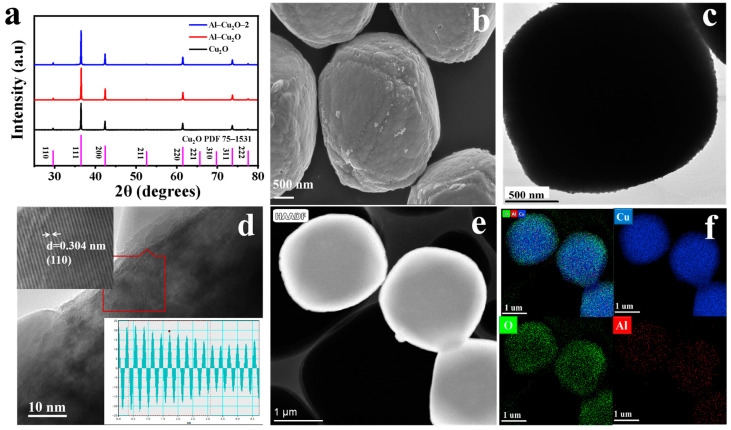
Characterization of Al–Cu_2_O: (**a**) XRD, (**b**) SEM, (**c**) TEM, (**d**) HRTEM, (**e**) HAADF–TEM, and (**f**) elemental mapping (blue, green and red represents Cu, O and Al element, respectively).

As shown in Figure 1, Al–Cu_2_O–X nanocrystals were prepared by a simple one-step method (Experimental section for details). To further characterize Al–Cu_2_O–X catalysts, the XRD pattern was used to study Cu_2_O, Al–Cu_2_O, and Al–Cu_2_O–2 crystal structure. From the XRD pattern in Figure 2a, the peaks at 29°, 36°, 42°, 61°, 73°, and 77° correspond to the (110), (111), (200), (220), (311), and (222) planes of Cu_2_O, respectively, which agree well with the octahedral Cu_2_O (PDF#75–1535). The XRD patterns of Al–Cu_2_O–X (3-6) are shown in Figure S1. During the preparation process, the catalyst was synthesized by adjusting the amount of Al^3+^, the concentration of NaOH, and the reaction time. Al–Cu_2_O–X (3-6) were all single-phase Cu_2_O nanocrystals. The morphologies of the Cu_2_O, Al–Cu_2_O, and Al–Cu_2_O–2 were monitored by scanning electron microscopy (SEM). Cu_2_O nanocrystals without Al doping showed an octahedron shape with a smooth surface (Figure S2a). Due to the doping effect of Al, the Al–Cu_2_O nanocrystal presented an octahedral shape with a more rough surface and formed a defect structure (Figure 2b), which may provide abundant active sites for CO_2_RR [36]. When the concentration of Al^3+^ increased from 0.02 M to 0.03 M, Al–Cu_2_O–2 catalyst exhibits a cube shape (Figure S2b). However, it was reported that the resulting cube Al–Cu_2_O catalyst is not conducive to forming C_2_H_4_ [27]. The better-performing Al–Cu_2_O with a homo-octahedral shape was observed by TEM (Figure 2c), which was consistent with the SEM image. The high-resolution transmission electron microscopy (HRTEM) image in Figure 2d presented that the lattice stripe spacing d marked was 0.304 nm, corresponding to the (110) crystal plane of Cu_2_O. The HAADF-STEM image (Figure 2e) also exhibited an octahedral shape. The composition of Al–Cu_2_O was reconfirmed by elemental mapping (Figure 2f). The Al (red), Cu (blue), and O (green) elements are uniformly distributed over the Al–Cu_2_O nanocrystals.

**Figure 3:**
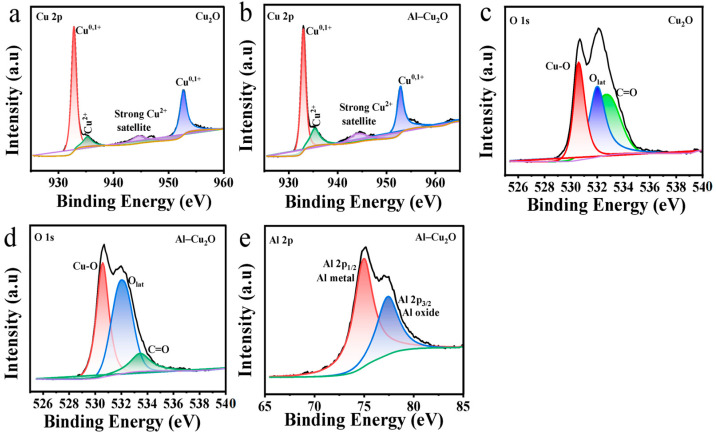
XPS spectrum of Cu 2p: (**a**) Cu_2_O and (**b**) Al–Cu_2_O; O 1s spectrum of (**c**) Cu_2_O and (**d**) Al–Cu_2_O, (**e**) Al 2p spectrum of the Al–Cu_2_O.

The surface composition and valence of Cu_2_O and Al–Cu_2_O nanocrystals were characterized by X-ray photoelectron spectroscopy (XPS). As shown in Figure 3a,b, four peaks were observed in Cu 2p spectrum for both Cu_2_O and Al–Cu_2_O samples. For Cu_2_O, the peaks at 932.78 and 952.62 eV corresponded to the binding energies of Cu 2p_3/2_ and Cu 2p_1/2_ of Cu_2_O or Cu, respectively. The binding energies at 935.28 eV and 944.48 eV were ascribed to the peaks of Cu^2+^. For Al–Cu_2_O, the 932.89 and 952.73 eV peaks corresponded to the Cu 2p_3/2_ and Cu2 p_1/2_ of Cu_2_O or Cu, respectively. The binding energy of 935.26 and 944.46 eV belonged to the peak of Cu^2+^. The above results showed that the existence of Cu^0^ may be due to the partial reduction of Cu_2_O in the CO_2_RR process [37]. The existence of trace CuO may be mainly due to the oxidation of a small amount of Cu_2_O catalyst to CuO in the air after the synthesis of Cu_2_O [38]. When octahedral Cu_2_O nanocrystals were doped with Al, the peaks of Cu 2p_3/2_, Cu 2p_1/2_, and Cu^2+^ of Cu_2_O or Cu were shifted positively. These results may be attributed to the introduction of Al, which can induce charge transfer from Al atoms to Cu atoms, thus modulating the electronic structure of Al–Cu_2_O. The existence of Cu_2_O was also confirmed in the O 1s XPS spectra of Cu_2_O and Al–Cu_2_O (Figure 3c,d). There were three XPS peaks in both catalysts, of which the peak at 530.5 eV corresponded to the Cu-O bond, and 532.11 and 532.77 eV corresponded to Olat and C=O, respectively [39]. In the high-resolution spectrum of Al 2p (Figure 3e), the peaks at 74.55 and 77.35 eV corresponded to the Al 2p_1/2_ and Al 2p_3/2_ of metal Al, respectively. The Al atom was 0.41% by XPS analysis, indicating that the Al–Cu_2_O catalyst has been successfully prepared.

### 2.2. Electrocatalytic CO_2_RR Performances

**Figure 4:**
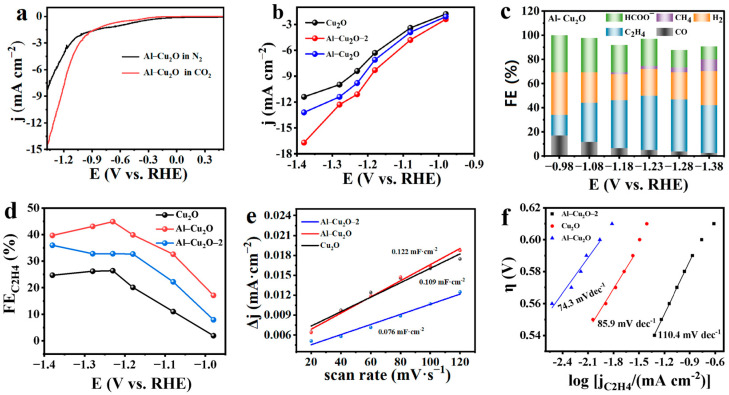
(**a**) LSV curves of Cu_2_O, Al–Cu_2_O and Al–Cu_2_O–2 catalysts in 0.1 M KHCO_3_ aqueous solutions saturated CO_2_, (**b**) partial current density of Cu_2_O, Al–Cu_2_O and Al–Cu_2_O–2 catalysts, sweeping speed of 5 mV s^−1^, (**c**) FE values of Al–Cu_2_O catalyst in 0.1 M KHCO_3_ aqueous solutions with saturated CO_2_, (**d**) The FE_C2H4_ values of Cu_2_O, Al–Cu_2_O and Al–Cu_2_O–2 catalysts, (**e**) The linear relationship between ΔJ and scanning rates, (**f**) Tafel plots of Cu_2_O, Al–Cu_2_O and Al–Cu_2_O–2.

To further analyze the electrochemical performance of the catalyst, the linear sweep voltammetry (LSV) of Cu_2_O and Al–Cu_2_O–X in saturated CO_2_ electrolyte and saturated N_2_ electrolyte were tested. The analysis of Figure 4a shows that the current density of Al–Cu_2_O catalyst in CO_2_ saturated electrolyte is higher than that in N_2_, indicating that Al–Cu_2_O catalyst had higher activity to CO_2_RR. The LSV curve was measured in a CO_2_-saturated 0.1 M KHCO_3_ electrolyte (Figure S3a). The current density of the Al–Cu_2_O catalyst in CO_2_ saturated electrolyte was higher than that of Cu_2_O and Al–Cu_2_O–2 catalysts, indicating that the Al–Cu_2_O catalyst had better electrocatalytic activity to CO_2_RR. Figure S3b shows the potentiostatic electrolysis of CO_2_ at various potentials. The almost constant current signal indicated that the Al–Cu_2_O catalyst exhibited good electrochemical stability during the CO_2_RR process. In Figure 4b, the formation rates of three kinds of catalysts were presented for ethylene products. The Al–Cu_2_O catalyst had a higher current density for ethylene formation than that of Cu_2_O and the Al–Cu_2_O–2 catalysts in a wide potential range. The partial current density of 16.7 mA cm^−2^ was achieved at −1.38 V (vs. RHE). The above results showed that the Al–Cu_2_O catalyst was more conducive to producing ethylene as the main product and has a better inhibitory effect on competition for hydrogen formation.

**Figure 5:**
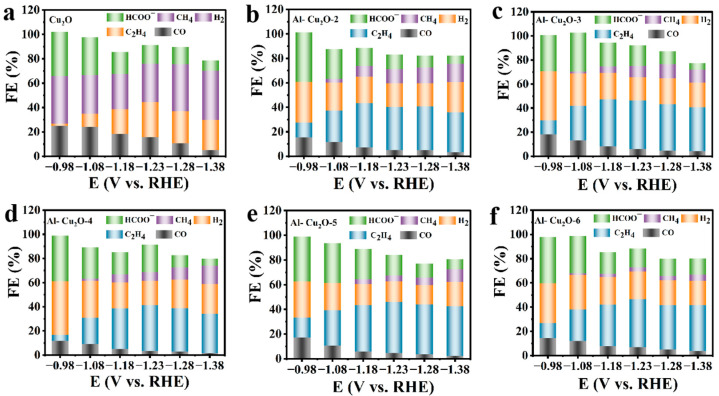
(**a**–**f**) FE values of Cu_2_O and Al–Cu_2_O–X (=2, 3, 4, 5, 6) catalysts in 0.1 M KHCO_3_ aqueous solutions with saturated gase CO_2_.

To determine the CO_2_RR selectivity of the Al–Cu_2_O catalyst, the reduction products were qualitatively and quantitatively analyzed. In this study, the reduction products of each catalyst were determined in the wide potential range from −0.98 V to −1.38 V (vs. RHE). From Figure 4c and Figure 5, the CO_2_RR products by Cu_2_O, and Al–Cu_2_O–X catalysts were C_2_H_4_, HCOO^−^, CO, CH_4_ and by-product H_2_. Figure 5a shows the FE of the electrochemical CO_2_RR product catalyzed by an octahedral Cu_2_O nanocrystal catalyst without the Al doping. The octahedral Cu_2_O nanocrystal catalyst had a good effect on inhibiting HER at low potential, and the FE_C2H4_ was 26.1%. As shown in Figure 4b, with the potential increase, the FE value of H_2_ decreases from 35.1% to 22.1%.

On the contrary, the FE value of C_2_H_4_ increases to 44.9% at −1.23 V (vs. RHE). The results showed the catalyst’s good selectivity for ethylene production and inhibition effect on HER. If an appropriate amount of Al (0.02 M) was introduced into the octahedral Cu_2_O nanocrystal (Figure 4c), the selectivity of the Al–Cu_2_O catalyst was improved. If more Al^3+^ was added to the reaction, The result showed that the FE of C_2_H_4_ was 32.8%, indicating that the catalyst had a good selectivity for ethylene (Figure 5b). we also studied the effects of catalysts synthetic conditions, including reaction time (Figure 5c,d) and NaOH concentration (Figure 5e,f), on the selectivity of the CO_2_RR product. The results indicated that optimising reaction time and NaOH concentration could give the catalyst a certain selectivity. Figure 4d compares the selectivity of three kinds of catalysts (Cu_2_O, Al–Cu_2_O, and Al–Cu_2_O–2) for ethylene products. Under different applied potentials, the efficiency of the Al–Cu_2_O catalyst for CO_2_RR to C_2_H_4_ was higher than that of the other two catalysts. This result suggested that the Al introduced into the catalyst affected the selectivity of the catalyst. This may be because Al-doped Cu_2_O will cause changes in the electronic structure and the morphology of the catalyst, thus reducing the adsorption energy of the catalyst for ethylene intermediates in the CO_2_RR process and enhancing the selectivity of the reaction to the products. It was worth noting that the Faradaic efficiencies sometimes do not reach 100%. A small number of liquid products may still be produced in the electrocatalysis process.

The electrochemical surface area (ECSA) is also a key point for the electrocatalyst. According to the formula for calculating ECSA, it is known that this parameter is related to the C_dl_ and C_ds_ values of their catalysts because the catalysts are coated on hydrophobic carbon paper (model 060). Therefore, the Cds of the three catalysts are the same, and only the C_dl_ value of the catalyst can be calculated to determine the ECSA of the catalyst. According the cyclic voltammograms (Figure S4a–c) of Cu_2_O, Al–Cu_2_O, and Al–Cu_2_O–2 catalysts at different scanning rates (20, 40, 60, 80,100, 120 mV s^−1^), the capacitance values of Cu_2_O, Al–Cu_2_O, and Al–Cu_2_O–2 catalysts were 0.109, 0.122, and 0.076 mF cm^−2^, respectively, as shown in Figure 4e. The largest C_dl_ of the Al–Cu_2_O electrocatalyst suggested the high electrochemical activity surface area of the Al–Cu_2_O–2 catalyst. This high ECSA can offer a lot of catalytic active sites for improving the electrocatalytic performance of CO_2_RR, which was consistent with the previous research conclusion.

The impedance of Cu_2_O and Al–Cu_2_O catalysts under open-circuit voltage was obtained (Figure S5). The EIS arc of the Al–Cu_2_O catalyst was smaller than that of the Cu_2_O catalyst. The results indicate that interface charges can be rapidly transferred during the reaction process, and catalytic activity can be improved. To better understand the activity and kinetics of Al–Cu_2_O materials on CO_2_RR, the Tafel slope analysis of the local current density of the catalyst product was carried out. As shown in Figure 4f, the Tafel slope of the Al–Cu_2_O catalyst (74.3 mV dec^−1^) was lower than that of Cu_2_O (85.9 mV dec^−1^) and the Al–Cu_2_O–2 (110.4 mV dec^−1^), indicating that the electron transfer rate of the catalyst is faster, which was beneficial to the rapid adsorption and desorption of the important intermediate from the surface of Al–Cu_2_O catalyst.

**Figure 6:**
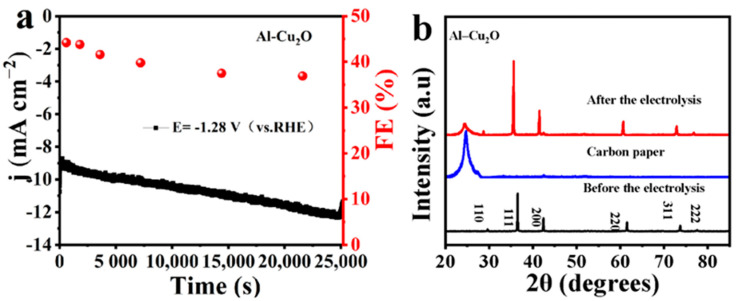
Al–Cu_2_O catalyst in 0.1 M KHCO_3_ electrolyte (**a**) electrochemical stability test pattern and (**b**) the XRD of Al–Cu_2_O catalyst after long–term stability test.

The stability of the Al–Cu_2_O catalyst was investigated in the CO_2_RR process. As seen in Figure 6a, the current density of the Al–Cu_2_O catalyst can be kept stable, and the FE of ethylene can be kept above 40% in the first 3600 s. With the change in reaction time, the current density increases gradually. However, the selectivity of the catalyst to ethylene decreased obviously after two hours of electrolysis. This may be because of the shedding of the catalyst in the long-term electrolysis process, resulting in a decrease in the FE of the catalyst. The stability of copper-based catalysts is poor. Therefore, other strategies must be used to improve the stability of copper-based catalysts for a long time [40]. The XRD pattern after long-term electrolysis showed that the Al–Cu_2_O showed good structure stability (Figure 6b) in the whole CO_2_RR test. After the electrolysis of the Al–Cu_2_O catalyst for 10 min, 20 min, 30 min and 7 h (Figure S6a–d), the morphology of the octahedron remains unchanged. With the increase of electrolysis time, some small pores appear on the catalyst’s surface. The appearance of these pores may provide more active sites, resulting in an increase in current density in the electrolysis process. However, it yielded a decrease in the FE of ethylene. The above results show that the catalyst can maintain stability under long-term electrolysis.

### 2.3. DFT Computations

**Figure 7:**
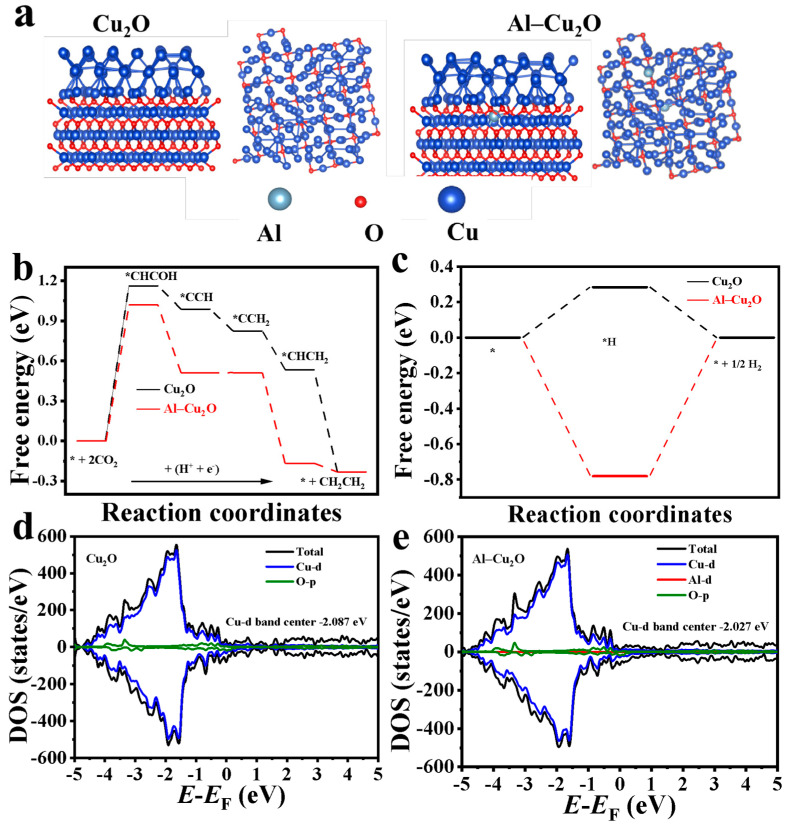
Free energy diagram of ethylene and hydrogen produced by CO_2_RR on the surface of (**a**) side and top views of Cu_2_O (001) and Al–Cu_2_O (001) configurations, (**b**) Cu_2_O (001) and (**c**) Al–Cu_2_O (001) catalysts, * reprents the active site; DOS of d orbitals on (**d**) Cu_2_O (001) and (**e**) Al–Cu_2_O (001) surfaces before *CHCOH adsorption.

We used Density functional theory (DFT) to further calculate, simulate and compare the CO_2_RR reaction path on the surface of Al–Cu_2_O and Cu_2_O catalysts to understand the path from CO_2_ to C_2_H_4_. Figure 7 shows the spatial structure (Figure 7a) and energy distribution of Al–Cu_2_O and Cu_2_O. Figure 7b shows the energy distribution of ethylene production and by-product H_2_ of Cu_2_O and Al–Cu_2_O catalysts. The Gibbs free energies of each intermediate along ethylene on Cu_2_O and Al–Cu_2_O catalysts *CHCOH, *CCH, *CCH, *CCH_2_, *CHCH_2_ (intermediates for ethylene production) and *H (intermediates to H_2_) have been calculated. Because the Gibbs free energy of the Al–Cu_2_O catalyst was lower than that of the Cu_2_O catalyst in each reaction step, the path of ethylene production of CO_2_RR was easier to occur. It can be seen that the strategy of doping Al to octahedral Cu_2_O was beneficial in improving the selectivity of product C_2_H_4_ [41]. At the same time, the analysis of Figure 7c showed that the Al–Cu_2_O catalyst with Al doping enhanced the adsorption of intermediate *H and further departed from the ideal hydrogen adsorption value (0 eV). It makes the competitive reaction of HER more disadvantageous. To further analyze the potential reason for the selective improvement of this product, the density of states (DOS) of d orbitals on Cu_2_O (001) and Al–Cu_2_O (001) surfaces before CHCOH adsorption was compared (Figure 7d,e). Since the electronic states near the Fermi level are mainly contributed by the d electrons of Cu atoms, it is observed that the reaction is mainly caused by the interaction between Cu and C, and the d band center of octahedron Cu_2_O (001) was −2.087 eV. The −2.027 eV of the Al–Cu_2_O (001) surface was closer to the Fermi level (0 eV), and the d-band shifts upward on the Abscissa, which makes the center of the d-band closer to the Fermi level and increases the density of electronic states. So, the adsorption of Cu atoms through d electrons and intermediates was facilitated, thus promoting the CO_2_RR process and improving the selectivity of the catalyst for the C_2_H_4_ product.

## 3. Materials and Methods

### 3.1. Preparation of Al–Cu_2_O Nanocrystals

The Al–Cu_2_O nanocrystals were synthesized with an improved method according to the literature [42]. The specific step was as follows: 10 mL of 0.6 M NaOH aqueous solution was first added to the sample bottle. Subsequently, a certain amount of CuCl_2_·2H_2_O, Al(NO_3_)_3_·9H_2_O and glucose were added to the sample bottle successively. The concentrations of CuCl_2_·2H_2_O, Al(NO_3_)_3_·9H_2_O, and glucose were 0.10 M, 0.02 M, and 0.07 M, respectively. After continuous agitation for 5 min, the sample bottle was placed in a 70 °C water bath and vigorously stirred for 4 min. The precipitation obtained by centrifugal collection was rinsed with deionized water and dried under vacuum at room temperature for 12 h to obtain an Al–Cu_2_O catalyst. At the same time, the effects of the amount of Al^3+^, NaOH concentration, and the sythiestic reaction time on ethylene products were also investigated in this chapter, and the optimum preparation conditions were obtained, as shown in the following Table S1.

### 3.2. Preparation of Al–Cu_2_O Coated Carbon Paper Electrode

5 mg of the prepared catalyst was added to 25 μL of Nafion. Then, 300 μL of distilled water and 175 μL of ethanol to prepare 500 μL of reagent was added and mixed by sonication for 2 h. Subsequently, 100 μL was uniformly applied with a pipette to a carbon paper (type 060) with a total surface area of 1 cm^2^. The loading on the carbon paper was calculated to be 1 mg cm^−2^ and dried in a vacuum oven to obtain the Al–Cu_2_O electrode for the next test.

### 3.3. Electrochemical Measurements

The electrocatalytic CO_2_RR was carried out in an H-type electrolytic cell with a proton exchange membrane (Nafion 117) separation. A platinum sheet (1 cm^2^) as the counter electrode and Ag/AgCl (saturated KCl) as the reference electrode, respectively. Before conducting the experimental test, CO_2_ (99.999% purity) or N_2_ gas was introduced into the electrolytic cell, which was saturated with 0.1 M KHCO_3_ (pH = 6.8) electrolyte after approximately 30 min. In this work, all electrochemical performance was measured on the electrochemical workstation (CHI760E, Shanghai Chenhua, Shanghai, China). All electrode potentials were converted into electrode potentials relative to RHE through the Nernst equation: E (vs. RHE)= E (vs. Ag/AgCl)+0.0591×pH+0.197 V. The electrochemical active surface area was tested by the cyclic voltammetry curves of the bilayer capacitance values at different scanning rates (20, 40, 60, 80, 100 and 120 mV s^−1^). The gaseous products were collected by electrolysis of the four catalysts in a 0.1 M KHCO_3_ electrolyte saturated with CO_2_ for 10 min at different measurement potentials and then analyzed using gas chromatography (8890, Agilent, Santa Clara, CA, USA). The liquid products of the four catalysts were collected by electrolysis in an aqueous 0.1 M KHCO_3_ solution saturated with CO_2_ for 30 min at each measurement potential, followed by qualitative and quantitative analysis using ion chromatography (AS-DV, Thermo Scientific, Waltham, MA, USA).

### 3.4. Product Analysis

The gas products are detected by gas chromatography (GC, Agilent 8890) directly from the gas outlet. The carbonaceous gas products from the cathode chamber are analyzed by a methane reformer and flame ionization detector (FID). A thermal conductivity detector (TCD) was used to detect the eCO_2_RR by-product H_2_. When the current stabilizes, the gas product is detected. Quantification of the gaseous products was determined by comparison with the standard curve. the Faraday efficiency (FE) of C_2_H_4_, H_2_ and CO was calculated as follows:

$$\mathrm{FE}=\frac{N\times n\times v\times F}{V_{m}\times j}\times 100\%$$

where  v is the CO_2_ flow rate (*v* = 20 mL min^−1^), n is the total molar fraction of C_2_H_4_, H_2_ or CO of the gas measured in the GC, *N* is the number of electrons required to form a molecule of H_2_ or CO (*N* = 2), *F* is Faraday’s constant (96,485 C mol^−1^), and *V_m_* is the molar volume of the gas at 298 K and *j* is current at each potential (A).

Liquid products Faraday efficiency test method: A saturated solution of electrocatalytic CO_2_ was electro-catalyzed by the Coulomb method using a controlled potential, and the electrolytic reduction product was analyzed and calculated after 0.5 h. The CO_2_ flow rate during electrolysis was controlled at 20 mL min^−1,^ and the liquid product was determined by ion chromatography (AS-DV, Thermo Scientific, Waltham, MA, USA). The FE of the liquid phase product was calculated as follows:

$$\mathrm{FE}=\frac{NnF}{Q}\times 100\%$$

where *N* is the number of electrons transferred, *n* is the amount of formate in the cathode chamber, *F* is Faraday’s constant (96,485 C mol^−1^) and *Q* is the total charge passing through the electrode.

## 4. Conclusions

In summary, the Al-doped octahedral Cu_2_O nanocrystal was successfully prepared and used as an efficient CO_2_RR electrocatalyst. The Al–Cu_2_O exhibited high activity and selectivity for ethylene production. The Al–Cu_2_O catalyst demonstrates a high % faradaic efficiency of 44.9% at −1.23 V (vs. RHE) for C_2_H_4_ production. The high catalytic activity for CO_2_ electrochemical reduction is due to the optimized electronic state by Al doping in octahedral Cu_2_O nanocrystals. The DFT simulation suggested the C–C coupling mechanism in the electrochemical CO_2_RR process. The Al–Cu_2_O doped Cu_2_O octahedron can significantly reduce the free energy in the coupling process of *CHCOH intermediate, promote the formation of C_2_H_4_, and inhibit the occurrence of HER side effect. Furthermore, our work demonstrates a simple doping strategy for preparing copper-based catalysts, which can be extended to the design and study of other highly efficient electrocatalysts.

## Data Availability

All data in this study can be found in public data bases and Supplementary Information, as described in the Material and Methods section (Section 3).

## Supplementary Materials

The following supporting information can be downloaded at: https://www.mdpi.com/article/10.3390/ijms241612680/s1. References [43,44,45,46] are cited in the supplementary materials.
