# Supplementary material for: Al-Doped Octahedral Cu2O Nanocrystal for Electrocatalytic CO2 Reduction to Produce Ethylene
Source: Int J Mol Sci. 2023 Aug 11;24(16):12680. doi: 10.3390/ijms241612680 (PMC10454826; doi:10.3390/ijms241612680)
Supplement: Supplementary file 1 [file ijms-24-12680-s001.zip › ijms-2523216-supplementary.pdf]

Supplementary Information for

Al-Doped Octahedral Cu_2O Nanocrystal for electrocatalytic CO_2 Reduction to produce Ethylene

Sanxiu Li[1,†], Xuelan Sha[1,†], Xiafei Gao, Juan Peng[1,*]

[1]State Key Laboratory of High−Efficiency Utilization of Coal and Green Chemical Engineering, College of Chemistry and Chemical Engineering, Ningxia University, Yinchuan 750021, China

Correspondence: pengjuan@nxu.edu.cn

[†]The authors contribute equally to this work.

Chemicals and Reagents

The following chemicals were purchased and used without further purification. Copper chloride dihydrate(purity≥99.9%, $CuCl_2·2H_2O$) powder was purchased from the Aladdin reagent network in China. Aluminum nitrate nine-hydrate (purity≥99.9%, $Al(NO_3)_3·9H_2O$), sodium hydroxide (purity≥99.9%, $C_6H_{12}O_6$), glucose (purity ≥99.9%, NaOH), potassium bicarbonate (purity ≥99.5%, $KHCO_3$) was purchased from Sinopharm Chemical Reagent Co., Ltd in China. Nafion solution (5.0 wt%) was obtained from Tianjin Incole Union Technology Co .,Ltd in China. Self-produced Deionized water. (18.24 MΩ cm^{-1}) was used in the entire experiment.

Characterization and measurement

Powder X-ray diffraction (XRD) measurements were conducted by a Smart Target X-ray diffractometer (Smart Lab, Japan). To observe the surface morphology of each electrocatalyst, we used an F-SEM cold field emission scanning electron microscope (F-SEM, Zeiss Sigma 300, Germany). High-resolution transmission electron microscope (HRTEM) and a high angle annular dark-field scanning transmission electron microscope (HAADFSTEM) was also used to obtain high-resolution images (FEI Talos F200x, America). Elemental content was analyzed by X-ray photoelectron spectroscopy (XPS, Thermo Scientific K-alpha, America), using a monochromatic Al-Kα radiation source (Mono Al-Kα) with 1486.6 eV of energy. Chemical states and surface material composition an X-ray photoelectron spectrometer was used for charge neutralization in the range of 75-150W with a low energy electron gun using a single cathode light source in a super-vacuum state. For the samples before electrocatalytic reaction, we directly characterize the freshly prepared electrodes. For the sample after electrocatalytic reaction, we washed the sample with deionized water, removed the surface electrolyte, and carried out characterization after drying.

Density Functional Theory Simulation

We carried out all the DFT calculations in the Vienna *ab initio* simulation (VASP5.4.4) code[1]. The exchange-correlation is simulated with PBE functional and the ion-electron interactions were described by the PAW method[2, 3]. The vdWs interaction was included by using empirical DFT-D3 method[4]. The Cu_2O (001) was heterocontacted with Cu (001) surface to simulate the catalysis of the reduction of CO_2 to CH_2CH_2. Atoms in the all the Cu (001) surface and upper two layers of the Cu_2O (001) surface are allowed to move freely while the bottom two layers of Cu_2O (001) surface are fixed to simulate the surface of structure. The Monkhorst-Pack-grid-mesh-based Brillouin zone k-points are set as 2×2×1 for all periodic structure with the cutoff energy of 400 eV. The convergence criteria are set as 0.01 eV A^{-1} and 10^{-5} eV in force and energy, respectively.

The free energy calculation of species adsorption (ΔG) is based on following model.

$$\Delta G = \Delta E + \Delta E_{ZPE} + \Delta H_{0\to T} - T\Delta S \quad (1)$$

Herein ΔE, ΔE_{ZPE}, and ΔS respectively represent the changes of electronic energy, zero-point energy, and entropy that caused by adsorption of intermediate. The $\Delta H_{0\to T}$ refers to the change in enthalpy when heating from 0K to T K.

Table S1 Preparation conditions of catalyst

Catalyst	The concentration of Al^{3+} (M)	The concentration of NaOH (M)	Time (min)	Remark
Cu_2O	0	0.6	4	
Al–Cu_2O	0.02	0.6	4	Optimum performance
Al–Cu_2O–2	0.03	0.6	4	
Al–Cu_2O–3	0.02	0.6	2	
Al–Cu_2O–4	0.02	0.6	6	
Al–Cu_2O–5	0.02	0.5	4	
Al–Cu_2O–6	0.02	0.7	4	

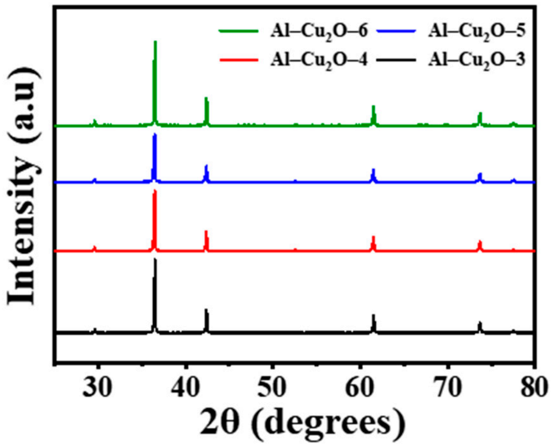

Figure S1 XRD patterns of Al–Cu_2O–X (X=3、4、5、6).

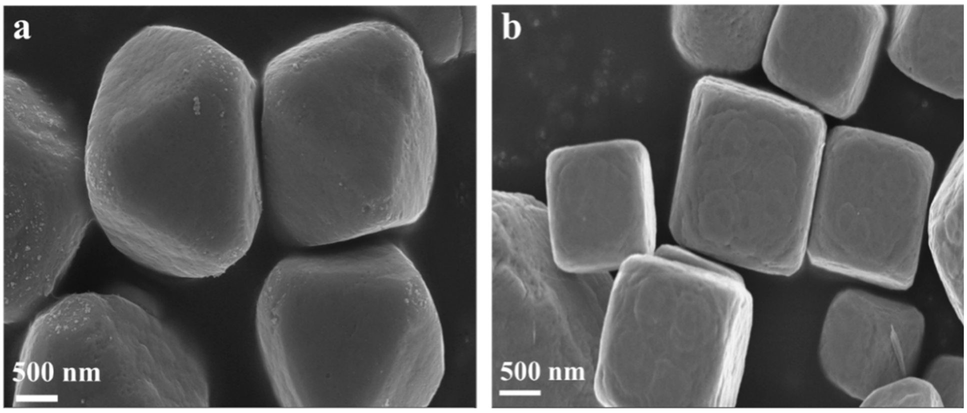

Figure S2 SEM images of (a) Cu_2O and (b) Al-Cu_2O-2.

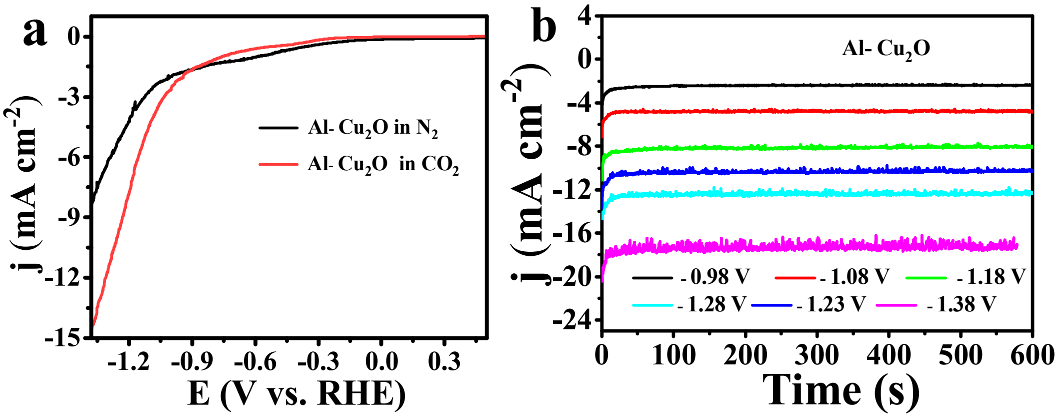

Figure S3 (a) The polarization curves of Al–Cu_2O catalyst in 0.1 M $KHCO_3$ aqueous solutions with saturated gases N_2 or CO_2 and (b) current response of Al-Cu_2O in 0.1 M $KHCO_3$ saturated with CO_2, sweeping speed of 5 mV s.

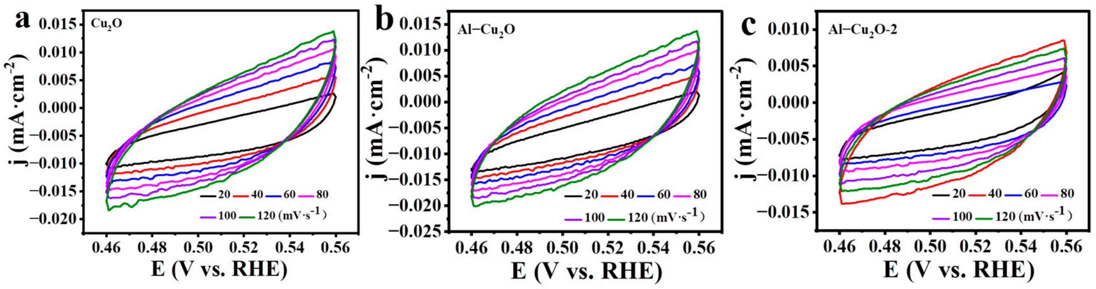

Figure S4 The catalyst of (a) Cu_2O, (b) Al–Cu_2O, and (c) Al–Cu_2O–2 volt–ampere curve in 0.1 M $KHCO_3$ aqueous solutions with saturated CO_2;

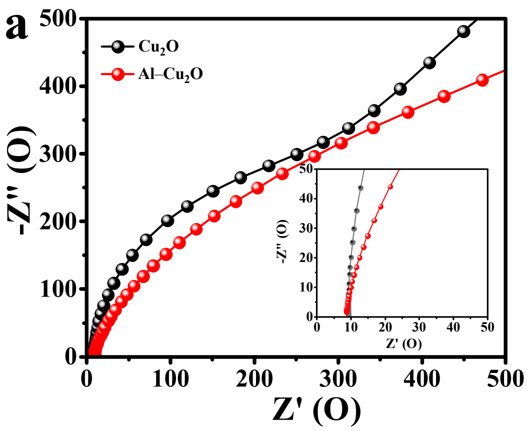

Figure S5 The EIS of Cu_2O and Al–Cu_2O catalysts.

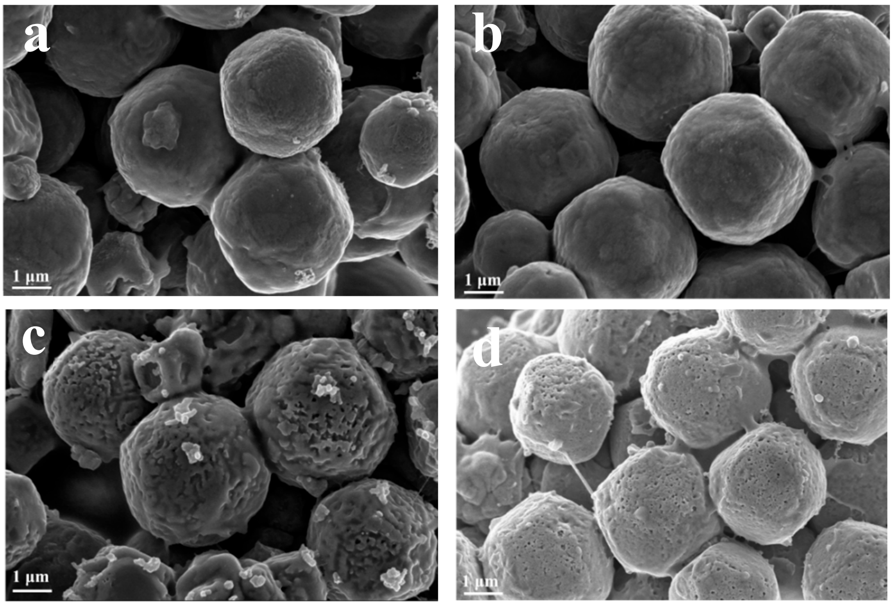

Figure S6 Al-Cu_2O catalyst in 0.1 M $KHCO_3$ electrolyte of Al-Cu_2O catalyst in 0.1 M $KHCO_3$ electrolyte of SEM image (a) 0 min, (b) 20 min, (c) 0 min and (d)7 h.

References

1. Kresse, G.; Furthmüller, J. J. Efficient iterative schemes for ab initio total-energy calculations using a plane-wave basis set. *Phys. Rev. B***. 1996,** 54, (16), 11169.
2. Perdew, J. P.; Burke, K.; Ernzerhof, M. J. Generalized gradient approximation made simple. *Phys. Rev. Lett.* **1996,** 77, (18), 3865..
3. Hammer, B.; Hansen, L. B.; Nørskov, J. K. Improved adsorption energetics within density-functional theory using revised Perdew-Burke-Ernzerhof functionals. *Phys. Rev. B* **1999,** 59, (11), 7413.

4. Grimme, S. J. Semiempirical GGA‐type density functional constructed with a long‐range dispersion correction. *J. Comput. Chem.* **2006,** 27, (15), 1787-1799.
